# Non-Markovian Complexity in the Quantum-to-Classical Transition

**DOI:** 10.1038/srep13353

**Published:** 2015-08-25

**Authors:** Heng-Na Xiong, Ping-Yuan Lo, Wei-Min Zhang, Da Hsuan Feng, Franco Nori

**Affiliations:** 1 Department of Physics and Center for Quantum Information Science, National Cheng Kung University, Tainan 70101, Taiwan; 2 Department of Applied Physics, Zhejiang University of Technology, Hangzhou 310023, China; 3 University of Macau, Taipa, Macau, China; 4 Center for Emergent Matter Science, RIKEN, Saitama 351-0198, Japan; 5 Physics Department, The University of Michigan, Ann Arbor, Michigan 48109-1040, USA

## Abstract

The quantum-to-classical transition is due to environment-induced decoherence, and it depicts how classical dynamics emerges from quantum systems. Previously, the quantum-to-classical transition has mainly been described with memory-less (Markovian) quantum processes. Here we study the complexity of the quantum-to-classical transition through general non-Markovian memory processes. That is, the influence of various reservoirs results in a given initial quantum state evolving into one of the following four scenarios: thermal state, thermal-like state, quantum steady state, or oscillating quantum nonstationary state. In the latter two scenarios, the system maintains partial or full quantum coherence due to the strong non-Markovian memory effect, so that in these cases, the quantum-to-classical transition never occurs. This unexpected new feature provides a new avenue for the development of future quantum technologies because the remaining quantum oscillations in steady states are decoherence-free.

The quantum-classical correspondence, searching for the unambiguous classical world starting from quantum theory^1^, is a time-honored question that has been explored since the birth of quantum physics. In the last two decades, significant progress has been made from the decoherence-dynamics point of view for systems interacting with their environments. A typical feature of decoherence is that the inevitable interaction between the system and its environment drives system states from the quantum regime (dominated by quantum coherence) to the classical regime (with a complete loss of quantum coherence). This process describes the quantum-to-classical transition (QCT)^2^. The environment-induced decoherence of quantum systems is not only a fundamental topic in quantum physics, but also the major obstacle in the development of quantum information processing. Many works, including both theories and experiments, have been devoted to this topic, see for examples Refs. 2, 3, 4. However, most of the previous works were focused on the weak system-environment coupling regime in which the memory effects or non-Markovian dynamics between the system and the environment are not essential. New experimental implementations of nanoscale solid-state quantum information processing^4^,^5^,^6^,^7^ make strong non-Markovian memory effects unavoidable. Thus the investigation of non-Markovian memory effects have recently become an important research topic in the study of quantum information processing^8^,^9^,^10^,^11^,^12^,^13^,^14^,^15^,^16^,^17^,^18^,^19^,^20^,^21^,^22^,^23^,^24^,^25^,^26^,^27^,^28^.

As we know, any realistic quantum system in nature has inevitable interactions with its surroundings (or environment). Such systems are called “open quantum systems”. All kinds of quantum devices, developed in recent years for quantum information processing, are open systems. In contract to an isolated quantum system, whose dynamics is governed by the Schrödinger equation, the dynamical evolution of an open quantum system is described by the master equation. Physicists have attempted for decades, using many different approaches, to derive the exact master equation for arbitrary open quantum systems. Unfortunately, no satisfactory answer was obtained, except for Brownian motion^29^,^30^,^31^,^32^. Very recently, by going beyond the toy model for Brownian motion, and using the coherent-state path-integral formalism^29^,^33^, an exact master equation for a large class of open quantum systems has been obtained^10^,^34^,^35^. These open systems consist of arbitrary 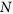 energy levels coupled, through various particle-particle exchanges (or particle tunnelings), to general environments with arbitrary continuous spectra at arbitrary temperature. These are also the basic physical systems widely used for recent developments of quantum transport and quantum decoherence in nano-electronics and nano-photonics^36^,^37^. The environment-induced energy-level renormalization, dissipation and thermal fluctuations are naturally incorporated into our master equation and can be nonperturbatively determined from the nonequilibrium Green functions^38^,^39^. These Green functions obey the microscopic Kadanoff-Baym equation for nonequilibrium dynamics^40^. Thus, we are able to take into account all the environment-induced non-Markovian memory effect in the study of the decoherence dynamics of nano-structured (nano-electronic and nano-photonic) quantum devices.

In this article, we present the general solution of the exact master equation for these quantum devices derived in Refs. 10,34,35. Based on the general solution, we demonstrate the complexity of the QCT for different dissipation processes. For a Markovian or a weakly non-Markovian environment, the system will eventually evolve into a thermal or a thermal-like state. This is the typical process for the QCT. For strongly non-Markovian environments, some of the quantum coherence could remain, leading to a quantum steady state or an oscillating quantum nonstationary state. In this case, the QCT never occurs. Some examples are presented to illustrate the complexity of non-Markovian dynamics in various different dissipation processes. At the end, we also present in detail the time evolution of Schrödinger cat-like states. The complexity of non-Markovian dynamics is examined, to demonstrate various scenarios for the QCT. An experimental proposal to measure such non-Markovian complexity in the QCT is also given.

## Results

### General Solution of Open Quantum Systems

We consider a large class of open quantum systems, consisting of arbitrary *N* energy levels, and coupling, via particle-particle exchange processes or particle tunnelings, to general environments with various possible continuous spectra. Such open systems correspond to the Fano-Anderson model in condensed matter physics^41^,^42^,^43^,^44^ and the Lee-Friedrichs model in atomic and molecular physics^45^,^46^,^47^. The exact master equation describing the time evolution of the reduced density matrix of such open systems was recently derived^10^,^34^,^35^
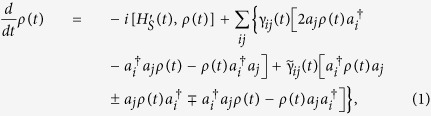
where 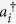 (*a*_*i*_) is the particle creation (annihilation) operator of the *i*th energy level of the open system. The first term in Eq. (1) describes the unitary evolution of the open system with the environment-induced renormalized Hamiltonian 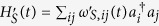. The second and third terms describe the environment-induced dissipation and fluctuations, respectively. The signs ± and 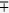 in the third term correspond to the system being bosonic or fermionic. This exact master equation nonperturbatively takes into account all the environment-induced non-Markovian memory effects, which is reflected by the renormalized frequency of the system 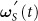, the dissipation coefficient **γ**(*t*) and the fluctuation coefficient 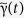. Here the environment is assumed initially in equilibrium at a finite temperature *T* and is initially decoupled from the system^29^,^48^.

All the time-dependent coefficients in the master equation are fully determined by the nonequilibrium Green functions through the following relations^10^,^34^,^35^,
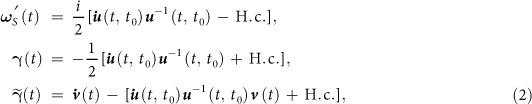
where the nonequilibrium particle propagating Green function ***u***(*t, t*_0_) and the fluctuated correlation Green functions 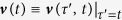 obey the Kadanoff-Baym equations
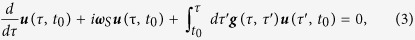

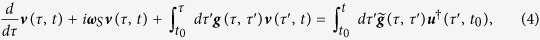
subjected to the boundary conditions ***u***(*t*_0_, *t*_0_) = 1 and ***v***(*t*_0_, *t*) = 0, with 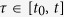. The frequency ***ω***_*S*_ is an *N* × *N* matrix for the *N*-level system, and the integral kernels, ***g***(*τ, τ*′) and 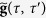, are defined by
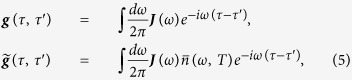
where ***J***(*ω*) is the spectral density which characterizes all the non-Markovian memory of the environment on the system. The function 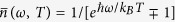 is the initial particle distribution of the environment.

The exact solution of these nonequilibrium Green functions has been presented in Ref. 20. Thus, by solving the exact master equation, the quantum state evolution of open systems can be precisely depicted. To be specific, consider a simple system with a single-particle energy level, e.g. a single-mode nanocavity coupled to a general environment with spectral density *J*(*ω*) at temperature *T*. Also, consider the system to be initially in an arbitrary state (in terms of the density matrix)
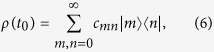
in which the diagonal matrix element *c*_*nn*_ represents the probability of the system in the particle number state 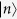, and the off-diagonal matrix element *c*_*mn*_(*m* ≠ *n*) characterizes the quantum coherence between the states 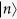 and 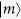. Then, under the influence of the environment, the time evolution of the quantum state, i.e. the exact solution of Eq. (1) is given by
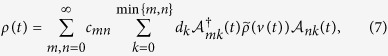
where
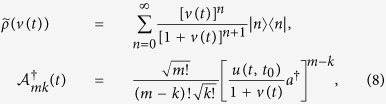


and 
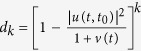
. These results show that the time evolution of the quantum state is fully determined by *u*(*t, t*_0_) and *v*(*t*). The analytical solution of these two basic nonequilibrium Green functions can be found in Ref. 20, and are also given in the Methods Section. As a result, the exact non-Markovian dynamics of open systems can be systematically explored within the present framework.

### Non-Markovian complexity of QCT

**Figure 1:**
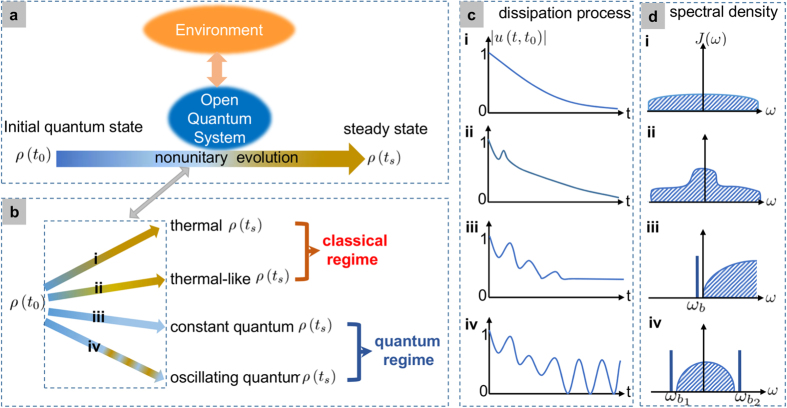
Schematic diagrams of the complexity of the quantum-to-classical transition. (**a**) An open quantum system, subject to an external environment, will undergo a nonunitary evolution. In this process, the quantum system may lose its initial quantum coherence, i.e., will exhibit quantum decoherence. This is the root of the quantum-to-classical transition (QCT). (**b**) For an arbitrary initial quantum state *ρ*(*t*_0_), a nonunitary evolution may lead the system to evolve into four possible steady states: thermal state, thermal-like state, quantum state, and oscillating quantum nonstationary state. If the system finally steps into the former two kinds of steady states, the QCT definitely happens. Otherwise, the system remains in the quantum regime, and the QCT (strictly) never emerges. The four kinds of steady states correspond to the four typical dissipation processes **i-iv** in figure (**c**) **i**, a dissipation process with exponential decay, where the non-Markovian memory of the environment is negligible. This is the standard QCT process; **ii**, a dissipation process in terms of a profile of exponential decay accompanied with short-time oscillations, which is a manifestation of weak non-Markovian memory; **iii**, a dissipation process stopped by a strong non-Markovian memory; **iv**, a dissipation process with oscillating steady state, also caused by a strong non-Markovian memory. The four dissipation processes stem from the four features of the spectral density (one-to-one correspondence) in figure (**d**) **i**, a wide-broad spectral density; **ii**, a spectral density which is a little different from the wide-broad one; **iii**, a spectral density with one forbidden frequency regime giving rise to a single localized mode *ω*_*b*_; **iv**, a spectral density with two (or more than two) forbidden regimes (band gaps) generating two (or more than two) localized modes 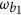 and 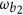.

From the equations (3) and (4), the particle propagating Green function *u*(*t, t*_0_) and the fluctuated correlation Green function *v*(*t*) are mainly determined by the spectral density of the environment *J*(*ω*), which is defined as the environment density of state 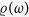 multiplied by the system-environment coupling strength *V*(*ω*), i.e., 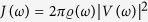. Based on our recent results^20^, different spectral densities for environments induce completely different dissipation and fluctuations on the system dynamics, due to different types of the non-Markovian memory effects. Therefore, from the general solution of the evolution state (7), one can derive the complexity of the QCT, as sketched in Fig. 1. Consider either weak or strong non-Markovian memories, arbitrary initial quantum states would be driven into steady states that could be thermal states, thermal-like states, quantum steady states, or oscillating quantum nonstationary states. Which scenario the system will eventually evolve is basically determined by the dissipation dynamics, described by the particle propagating function *u*(*t, t*_0_) of the system. While the fluctuation dynamics, given by the fluctuated correlation *v*(*t*), determine the nonequilibrium particle distributions. Below we will discuss case-by-case the four different scenarios.

#### 1. Thermal steady states

If a system undergoes a dissipation process with a monotonic exponential decay, as shown in Fig. 1c.i, i.e.,
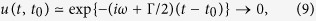
when *t* → *t*_*s*_ ≫ 1/Γ, then the system will eventually lose all the initial quantum coherence and evolve into a mixed steady state of the form
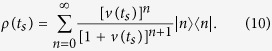


This process happens usually when the system-environment coupling is sufficiently weak (compared with the system frequency) and the spectral density has a rather broad distribution, *J*(*ω*) ≃ Γ, as shown in Fig. 1d.i. Correspondingly, the particle correlation in the environment gives almost a delta-function profile in the time domain. In other words, the system loses all the memory during its evolution, due to rapidly exchanging information with its environment. Thus, the initial quantum coherence is completely washed away at the end [so the steady solution (10) is totally independent of the initial state (6)]. On the other hand, the wide-broad spectral density ensures that the environment remains in its initial thermal equilibrium state, and the system finally reaches equilibrium with its environment. The particle correlation in the steady state becomes
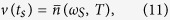
and the solution (10) is indeed the well-known thermal state at the given temperature *T*. This is the microscopic picture for the QCT that has been extensively investigated^2^,^30^.

#### 2. Thermal-like steady states

However, for many practical open systems, their dynamics do not follow a monotonic exponential decay, in particular, when the system-environment couplings become a bit stronger, or the reservoir has band gaps. The corresponding dissipation dynamics usually shows a nonexponential decay and sometimes even becomes dissipationless^20^. Therefore, the quantum state may not evolve into the thermal state (10) as could be naively expected. To be specific, let us now consider a spectral density, Fig. 1d.ii, which is a bit different from the wide-broad distribution shown in Fig. 1d.i. The particles in the environment take a correlation time that is not extremely short in comparison with the time scale of the system. In this case, the system will partially remember (memory) its response to the information exchange with the environment in a similar time scale. Consequently, the dissipation process exhibits a short-time deviation from the exponential decay, although *u*(*t*_*s*_, *t*_0_) → 0 at the end, see Fig. 1c.ii. This is a manifestation of the weak non-Markovian memory effect. The quantum state of the system will reach the steady state with the same form as Eq. (10) but the particle correlation resulted from thermal fluctuations becomes
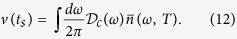


Notice that the memory effect in this case takes place in a relatively short time, so that the steady-state solution is still independent of the initial state (6). The quantum coherence is also totally lost at the end. But the steady-state particles in the system are thermally distributed in the broadened spectrum of the system,
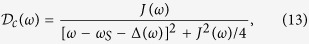


arisen from the coupling to the environment^20^, where 
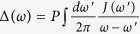
. If *J*(*ω*) is approximately a constant, then Δ(*ω*) → 0, and Eq. (12) is reduced to Eq. (11) of the case 1. We name the steady state of Eq. (10) with the distribution (12) as a thermal-like state. We will show later that such a weak non-Markovian memory effect is achieved when the system-environment coupling strength is smaller than a critical value for some colored spectral densities.

#### 3. Quantum steady states

The situation becomes rather interesting when the spectral density does not cover the whole frequency regime, as shown in Fig. 1d.iii. In this case, the system has a localized mode *ω*_*b*_. As shown in Fig. 1c.iii, the dissipation dynamics is significantly different from that shown in Fig. 1c.i and ii. In particular, the dissipation is even stopped after a certain time (becomes dissipationless, and *u*(*t*_*s*_, *t*_0_) remains a nonzero constant). This is a striking manifestation of the strong non-Markovian memory effect. The quantum state of the system will evolve into the steady state
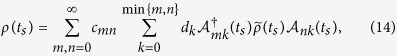
which has exactly the same form as the general solution of Eq. (7), with the steady-state solution of the particle propagating Green function
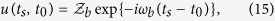


obtained directly from Eq. (31) in the Methods, where 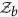 is the amplitude of the steady-state particle field 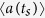 arisen from the localized mode^20^. The particle correlation is characterized by
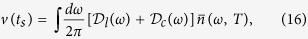


which is similar to Eq. (12) in the case 2, but having an additional contribution arose from the localized mode, 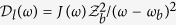. This shows that the solution (14) never becomes a completely mixed state as long as the dissipation is stopped at a certain time (i.e., *u*(*t*_*s*_, *t*_0_) ≠ 0 due to the existence of the localized state). The off-diagonal matrix elements in the state (14) never vanish so that some quantum coherence is constantly maintained. Also, the average particle number in the system is given by
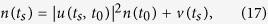


which depends explicitly on the initial particle number^15^,^23^,^25^. In other words, the system has the longest memory effect (memorizes forever some of its initial state information), and never reaches equilibrium with its environment. This indicates that open systems can stay in a steady but nonequilibrium state that has constant information (coherence) exchange with its environment through non-Markovian memory processes.

#### 4. Oscillating quantum steady states

Moreover, when the spectral density has more than one band gap, such as quantum dots in semiconductor nanostructures and micro/nano-cavities in photonic crystals, it could bring more than one localized state to the system, as shown in Fig. 1d.iv. These localized modes produce dissipationless dynamics of the system with different oscillation frequencies. As a result, the system state evolves into a steady state having the same form as Eq. (14) but the dissipationless dynamics is very different. It combines different localized modes to forming an oscillating steady state with
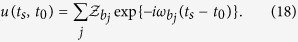
As a result, the particle correlation, still given by Eq. (16) in which 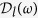 becomes
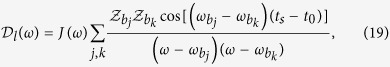
oscillates in time. This pure quantum oscillation is a very interesting feature for quantum devices. It may be useful for quantum information processing against decoherence, because the localized modes have already taken into account all the environmental back-action effects and therefore are decoherence-free. A typical physical realization for this situation is a quantum device coupled to a structured reservoir, as we will demonstrate below.

In summary, we prove that for a general non-Markovian environment, the system can evolve into one of four possible steady states: thermal state, thermal-like state, quantum steady state, and oscillating quantum nonstationary state. The first two correspond to the standard QCT usually occurring in the weak-coupling limit, while the last two steady states partially maintain the initial-state quantum coherence, which is a remarkable manifestation of the strong non-Markovian memory effect. Thus, the quantum-to-classical transition due to the environment-induced decoherence is crucially determined by the system-environment coupling strengths as well as the environment spectral properties. This transition mainly occurs when the system-environment coupling strength is not so strong and/or the environment does not contain band gap structures such that the interactions between the system and its environment cannot generate localized bound states. The localized bound states do not allow the system to approach equilibrium with its environment^23^,^25^,^41^.

Note that the classical states in the above QCT refer to completely mixed states in which all quantum coherence effects are lost through the decoherence process, as defined explicitly in the Introduction (also see^2^). This classical domain is different from the conventional classical limit of an isolated quantum system. The latter requires to find some intrinsic parameters (such as energy scale, particle number or angular momentum, etc.) of the system, such that certain semiclassical approximation can be made under a proper limit of these parameters with respect to the Planck constant^1^. However, the conventional classical limit can be explored in the above QCT in the weak system-environment coupling at high temperatures. Note that the general solution of the fluctuation Green function of Eq. (4), given by Eq. (33), is the generalized nonequilibrium quantum fluctuation-dissipation theorem^20^. In the weak system-environment coupling regime, the localized bound states either do not occur, as we have shown above, or their contribution to the fluctuation dynamics is generally negligible when the environment has band-gap structures^25^. Thus, the generalized nonequilibrium quantum fluctuation-dissipation theorem of Eq. (33) is reduced to the equilibrium fluctuation-dissipation theorem^49^ at the steady-state limit (see the detailed proof in the Supplemental Material of Ref. 20). Then at high temperatures, the equilibrium fluctuation-dissipation theorem is reduced further to the Einstein’s fluctuation-dissipation theorem, which can also be derived from the classical Langevin equation^50^. This classical limit is consistent indeed with the result of the classical Brownian motion derived by Caldeira and Leggett^30^ from a quantum harmonic oscillator coupled to a thermal bath within the same framework.

### Applications

We now illustrate how the complexity of non-Markovain dynamics can be observed in real physical systems. Since the steady state that an open system will eventually reach depends closely on features of the spectral density, we consider first a general environment with the Ohmic-type spectral density^48^ for simulating a large class of thermal reservoirs,
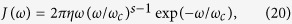
where *η* is a dimensionless system-environment coupling strength, and *ω*_*c*_ is a cutoff frequency. The parameter *s* classifies the spectral density into three types: sub-Ohmic (0 < *s* < 1), Ohmic (*s* = 1), and super-Ohmic (*s* > 1). These describe different non-Markovian environments for different physical systems.

**Figure 2:**
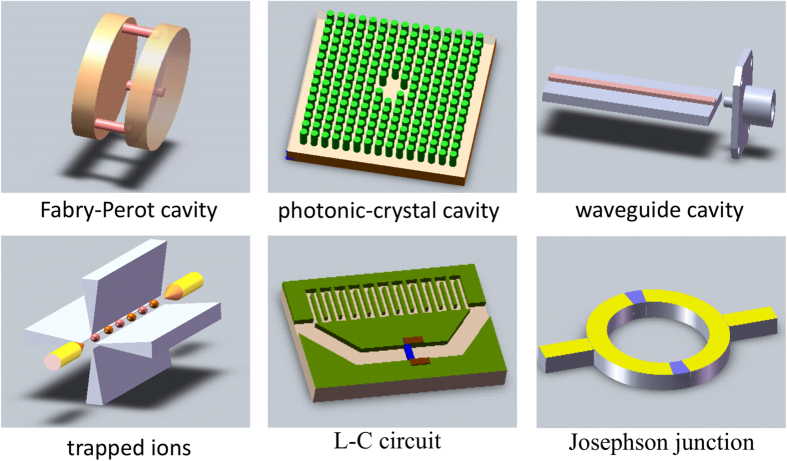
Illustration of various examples of open systems. An open photon system with single-mode can be any kind of cavity, such as a Fabry-Parot cavity, a photonic-crystal cavity, or a waveguide cavity. It could also be trapped ions with a vibrational mode. Alternatively, it could be various solid-state devices as well; for instance: *LC* circuits and Josephson junctions.

The noise process of some examples listed in Fig. 2 can indeed be described by the Ohmic-type spectral density of Eq. (20). In particular, for solid-state devices at low temperatures, such as nano-scale *LC* circuits, superconducting Josephson junctions, and quantum dot devices, the dominant noise sources could be simulated by sub-Ohmic spectral densities or 1/*f* low-frequency noises^51^,^52^. For nanomechanical resonators, the interesting noise source should be phonon-bath noises which are mainly described by super-Ohmic spectral densities^48^,^53^. However, for photonic crystal cavities and waveguide cavities, the spectral densities may contain photonic band gap structures^25^,^54^. These different physical systems could provide very different non-Markovian decoherence behaviors under different noise processes, as we show below.

**Figure 3:**
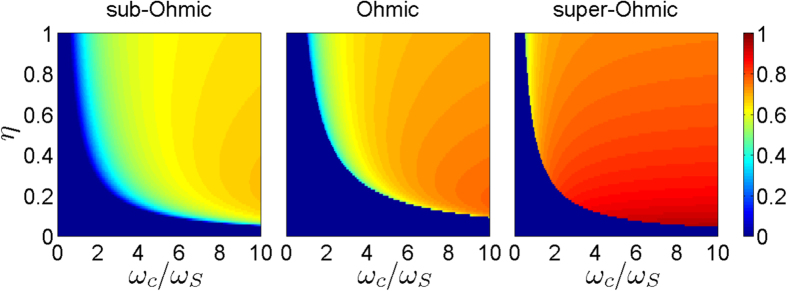
Transition from thermal-like state and quantum steady state. The figure displays the steady value of the particle field amplitude |*u*(*t*_*s*_, *t*_0_)| with sub-Ohmic (*s* = 1/2), Ohmic (*s* = 1), and super-Ohmic (*s* = 3) spectral densities for arbitrary system-environment coupling strength *η* and cutoff frequency *ω*_*c*_. Here *ω*_*S*_ is the frequency of the system. For each type of spectral density, |*u*(*t*_*s*_, *t*_0_)| remarkably transits from zero to nonzero with the boundary line *η*_*c*_ = *ω*_*S*_/[*ω*_*c*_Γ(*s*)], corresponding to the transition from classical thermal-like steady state to quantum steady state, respectively.

Any Ohmic-type spectral density takes the profile shown in Fig. 1d.iii, with *ω* ≥ 0. It shows that there is a localized mode with frequency *ω*_*b*_ located in *ω* < 0 when the coupling strength exceeds the critical point *η*_*c*_ = *ω*_*S*_/[*ω*_*c*_Γ(*s*)], where 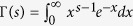 is the gamma function. This negative but finite renormalized frequency *ω*_*b*_ is a natural result of the open system in the strong system-reservoir coupling regime for Ohmic-type spectral densities^20^,^23^. Although the renormalized frequency of the system is negative, it is easy to prove that the total energy is always positive-definite for all decoupled initial states^48^ used for deriving the exact master equation. This is mainly because of the conservation of the total particle number for the total system. The boundary line *η*_*c*_ = *ω*_*S*_/[*ω*_*c*_Γ(*s*)] distinguishes the dissipation and dissipationless regimes (through the zero and nonzero values of the particle field amplitude |*u*(*t*_*s*_, *t*_0_)|), which tells how and when a thermal-like state or a quantum steady state will show up through varying the coupling strength in the spectral density, as shown in Fig. 3. Note that the standard QCT with the state (10) known in the literature corresponds to the limit case where the height of the spectral distribution *ηω*_*c*_/*ω*_*S*_ ≪ 1 and the spectral width *ω*_*c*_/*ω*_*S*_ ≫ 1, in which the non-Markovian memory is truly negligible.

To demonstrate the existence of oscillating quantum steady states, we consider a nanocavity subject to a structured reservoir consisting of a coupled-resonator array^17^,^54^. Thus, the reservoir can be modeled by a tight-binding one-dimensional system, and its dispersion has the form *ω*_*k*_ = *ω*_*c*_ − 2*ξ* cos (*k*). This leads to the finite-band structure, for which the spectral density
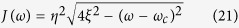


with the band: |*ω* − *ω*_*c*_| ≤ 2*ξ*^17^,^54^, where *ω*_*c*_ is the band center of the reservoir, *ξ* is the intercavity coupling inside the reservoir, and *η* is the ratio between the cavity-reservoir coupling and intercavity coupling *ξ*. It is easily to show that, when the coupling strength 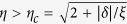, where *δ* = *ω*_*S*_ − *ω*_*c*_ is the detuning, there are two localized modes 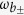 located at two outsides of the band. These two simultaneously-occurring modes cause the oscillatory dissipation dynamics, as shown in Fig. 1d.iv. In particular, in the zero-detuning case, the steady value of the particle field oscillates as a cosine:
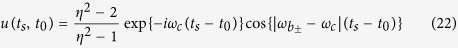
with two localized modes 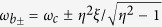. This gives rise to an oscillating quantum nonstationary state.

### The quantum-to-classical transition for Schrödinger cat-like states

We now examine the time-evolution of Schrödinger cat-like states in a general environment. Schrödinger cat-like states have been considered as a prototypical example used to demonstrate the QCT^3^,^4^. Here we shall demonstrate the memory-induced complexity of the non-Markovian dynamics through the measurement of the remaining quantum coherence in the steady states of Schrödinger cat-like states, in terms of the fringe visibility.

A Schrödinger cat-like state is defined as a superposition of two oppositely-moving coherent states: 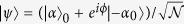. Its time evolution at a later time *t* is given by
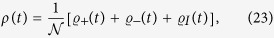
where
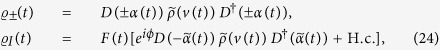
and 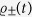 and 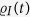 correspond to the evolution of the coherent states 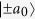 and the interference between them, respectively. Here *D*(*α*) is the displacement operator, and *α*(*t*) = *u*(*t, t*_0_)*α*_0_ and 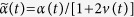. The prefactor *F*(*t*) in the interference term 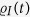 is just the fringe visibility, given explicitly in (27).

The Wigner distribution gives a description of the density matrix in phase space (in the coherent-state representation here). For the quantum state (23), the corresponding Wigner distribution is given by^17^
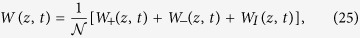
where
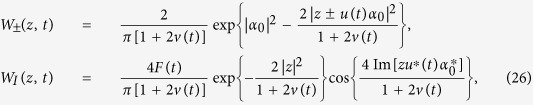
and *W*_±_(*z, t*) and *W*_*I*_(*z, t*) are the Wigner distributions of 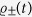 and 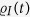, respectively.

The fringe visibility is an experimentally-measurable signature of quantum conference, i.e. interference, for identifying the quantumness of a density matrix. It can be defined as the peak-to-peak ratio between the interference and the direct terms in the Wigner function for Schördinger cat-like states^2^. From the Wigner distribution of Schrödinger cat-like states, the fringe visibility is exactly given by the prefactor in the interference term 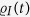,
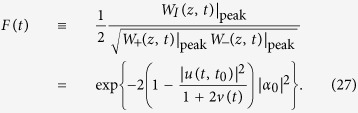


Since the fringe visibility ranges from the minimum value *F*_min_ = exp[−2|*α*_0_|^2^] (complete decoherence) to the maximum value *F*_max_ = 1 (full coherence), the decay of *F*(*t*) directly depicts the quantum decoherence process. For weak non-Markovian memory processes, the particles in the system will be eventually dissipated into the environment [because *u*(*t*_*s*_, *t*_0_) → 0] so that the fringe visibility reaches its minimum value. That is, quantum coherence is completely lost, and the system evolves into the classical regime with a thermal or a thermal-like steady state^3^,^4^. However, if there are band gaps appearing in the spectral density, then a strong non-Markovian memory effect leads to dissipationless dynamics^20^, characterized by a nonzero steady-state value of *u*(*t*_*s*_, *t*_0_). Thus the initial quantum coherence in the Schrödinger cat-like state can be partially maintained after the system reaches its steady state, which can be measured through the fringe visibility with the value greater than its minimum.

**Figure 4:**
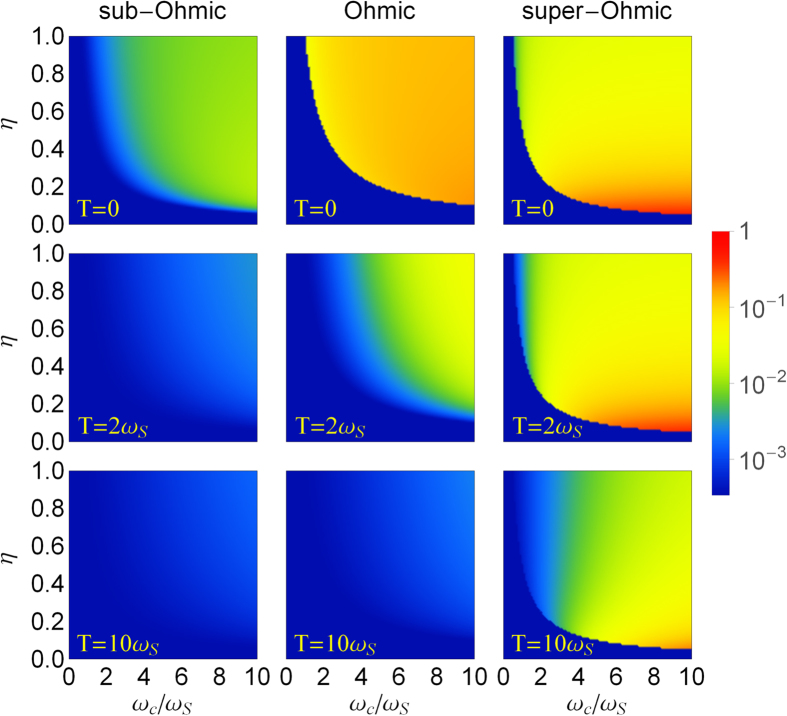
The fringe visibility of Schrödinger cat-like states. The fringe visibility *F*(*t*_*s*_) of an initial Schrödinger cat-like state for three different Ohmic-type spectral densities: sub-Ohmic, Ohmic, and super-Ohmic at zero and finite temperatures (*T* = 0, 2*ω*_*S*_, 10*ω*_*S*_). The initial coherent amplitude is *α*_0_ = 2.

Figure 4 shows the steady-state values of the fringe visibility for Ohmic-type spectral densities at zero and finite temperatures, respectively. In the regime *η* < *η*_*c*_, *F*(*t*_*s*_) takes only its minimum value *F*_min_ ~ 0 (for *α*_0_ = 2). This corresponds to the classical steady-state regime. While for *η* > *η*_*c*_, *F*(*t*_*s*_) is always greater than *F*_min_. Namely, partial quantum coherence is maintained, and the corresponding steady states always memorize some of the initial quantum coherence information, and thus the system never really reaches the classical regime. The difference between *F*(*t*_*s*_) and *F*_min_ measures how much quantum coherence remains in the steady state. Obviously, at finite temperatures, the remaining quantum coherence in the regime with *η* > *η*_*c*_ is partially suppressed by thermal fluctuations, in comparison with the zero temperature case. When the temperature becomes higher, the memory effect decreases rapidly. In other words, thermal fluctuations speed up the transition from the quantum domain to the classical domain, and the memory effect becomes in general less important in the high temperature regime, as expected. This result agrees with the recent work by Pachón *et al.*^55^. Also notice that for a super-Ohmic reservoir (*s* = 3), the remaining quantum coherence does not change too much for different temperatures. This is because for a super-Ohmic spectral density with *s* > 2, the damping rate is extremely small, even in the weak system-reservoir coupling regime^28^,^56^, while the damping quickly stops in the strong-coupling regime because of the occurrence of the localized bound states^28^. Thus, thermal fluctuations cannot suppress the non-Markovian memory effect, as shown in Fig. 3. In other words, the non-Markovian memory effect is robust against thermal fluctuations in preserving quantum coherence for super-Ohmic environments.

**Figure 5:**
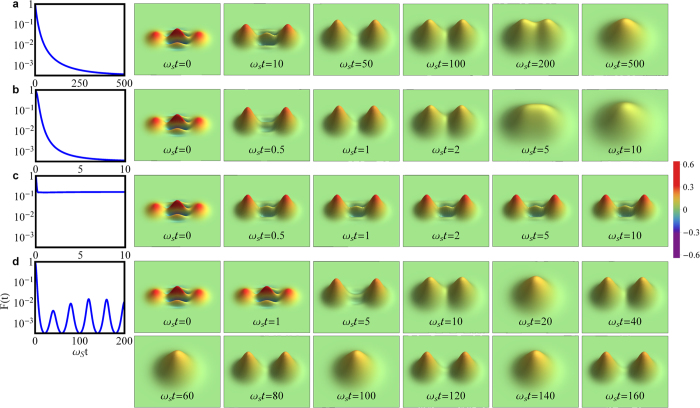
Dynamics of the Fringe visibility and Wigner distribution. On the left column, the dynamics of the fringe visibility *F*(*t*) is displayed. On the right column, several snapshots of the Wigner distribution are shown for the time-evolution. The four dynamical processes here correspond to the four kinds of dissipation processes in Fig. 1c. Here the parameters are set as: (**a**) Ohmic spectral density with coupling strength *η* = 0.001 and cutoff frequency *ω*_*c*_ = 10*ω*_*S*_. The Markovian memory is almost negligible; (**b**) Ohmic spectral density with coupling strength *η* = 0.1 and cutoff frequency *ω*_*c*_ = 5*ω*_*S*_. Here *η* < *η*_*c*_ = 0.2. This is still in the weak non-Markovian memory regime; (**c**) Super-Ohmic spectral density with coupling strength *η* = 0.12 and cutoff frequency *ω*_*c*_ = 5*ω*_*S*_. Here *η* > *η*_*c*_ = 0.1 and the effect of the strong non-Markovian memory shows up; (**d**) The spectral density for a tight-binding one-dimensional system with coupling strength *η* = 3.0 and band center *ω*_*c*_ = *ω*_*S*_. Here the region 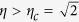 is in the strong non-Markovian memory regime. In all of these four cases, the initial environment temperature is set as *T* = 2*ω*_*S*_ and the initial coherent amplitude *α*_0_ = 2.

To see how fast the coherence is lost in different dissipation processes, we compute the time dependence of the fringe visibility. In Fig. 5, the time-dependence of the fringe visibility is plotted, with the corresponding time evolution of the Wigner function for an initial Schrödinger cat-like state for all four typical dissipation processes. The initial fringe visibility takes always the maximum value *F*(*t*_0_) = 1, namely, the cat state has the maximum quantum coherence. The initial Wigner function always has negative interference fringes (see the figures at *t*_0_ = 0), as a signature of quantumness. In the Markovian (memory-less) process, the initial cat state smoothly loses all its quantum coherence and becomes a thermal state (the Wigner function becomes a thermal Gaussian distribution located at the origin, see the last plot in Fig. 5a). Correspondingly, the fringe visibility *F*(*t*) decays exponentially from 1 to *F*_min_. For other non-Markovian memory processes, the initial cat state takes different decoherence behaviors. For a weak non-Markovian decay given in Fig. 5b, although the steady state (reached at *t* = 10/*ω*_*S*_) is a thermal-like one, its decoherence is much faster than the pure thermal state (which takes *t* = 500/*ω*_*S*_ to reach the steady state as shown in Fig. 5a), so does the fringe visibility. Furthermore, with a strong non-Markovian memory effect, see Fig. 5c (or 5d), the fringe visibility only decays to a constant value higher than *F*_min_ (or a steady oscillation pattern), where the Wigner function never becomes a thermal Gaussian distribution. In either case, the dynamics of the fringe visibility clearly characterizes the decoherence process of the system.

### An experimental proposal for measuring the non-Markovian memory effects

Decoherence processes have been measured through the reconstruction of the density matrix^3^,^57^. Here, we propose an alternative and simpler method to experimentally measure the fringe visibility. The fringe visibility given by Eq. (27) is fully determined by the particle propagating function |*u*(*t, t*_0_)|^2^ and the fluctuation correlation function *v*(*t*). These two Green functions provide full information of dissipation and thermal fluctuation of the system induced by the environment, and are independent of the initial state *ρ*(*t*_0_) of the system [see Eqs (3) and (4)]. Meanwhile, the particle number (intensity) of the system is *n*(*t*) = |*u*(*t, t*_0_)|^2^*n*(*t*_0_) + *v*(*t*), which can be directly measured. Thus one can obtain |*u*(*t, t*_0_)|^2^ and *v*(*t*) by detecting the intensity (particle number) under two different initial states. In cavity systems, the intensity detection is a well-developed technique, so the fringe visibility can be directly measured without going through the reconstruction of the density matrix^3^,^57^.

For example, we consider two initial coherent states (two laser beams) with different intensities *n*_1_(*t*_0_) and *n*_2_(*t*_0_). Then by detecting the intensities *n*_1_(*t*) and *n*_2_(*t*) at time *t*, one can obtain the Green functions |*u*(*t, t*_0_)|^2^ and *v*(*t*) in the time domain as follows
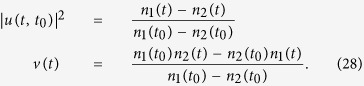


In this manner, not only the dissipation and fluctuations, characterized explicitly by *u*(*t, t*_0_) and *v*(*t*), and also the fringe visibility *F*(*t*) can be easily obtained. It is also worth pointing out that once the Green functions *u*(*t, t*_0_) and *v*(*t*) can be obtained through the detection of the intensity, one can extract the spectral density from the Fourier transformation of these Green functions. Explicitly, the Fourier transformation, 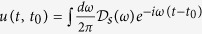, provides the modified spectrum of the system due to the coupling to the environment^20^:
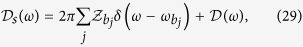
where 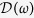 is given by Eq. (13) as a function of the spectral density *J*(*ω*). This could provide an alternative, but simpler, experimental approach to measure the spectral density *J*(*ω*), in comparison to the direct measurement of the noise spectrum from the two-time correlation functions of the relevant physical quantities^51^,^52^,^58^.

## Discussions

We have provided a general picture on the QCT for quantum systems coupled to arbitrary non-Markovian environments. We find that a Markovian (or weak non-Markovian) environment will give rise to a thermal state (or a thermal-like state) of the quantum system. That is, the system finally loses all its initial quantum coherence, which produces the expected QCT phenomenon. However, a strong non-Markovian environment could maintain partial or full quantum coherence, leading to a quantum steady state or an oscillating quantum nonstationary state in which the QCT never occurs. This brings up totally new features for quantum devices. Namely, a quantum device will not always evolve into equilibrium with its thermal reservoir, and it can maintain quantum oscillations in its localized modes. These unexpected new features provide a new avenue for the development of future quantum technologies because the remaining quantum oscillations in steady states are decoherence-free.

Taking Schrödinger cat-like states as specific initial states, we explicitly show how to employ the fringe visibility to quantitatively measure the dynamical quantum coherence in the steady state, to demonstrate the non-Markovian complexity in various decoherence processes. The results not only reproduce the QCT through the decoherence of Schrödinger cat-like states, which has been measured in Haroche’s experiments^3^, but also provide new features of non-Markovian decoherence for structured reservoirs that could be measured in the future. We also propose how to experimentally measure the fringe visibility through the measurement of the particle number of the system. This experimental scheme provides an easily-accessible way to observe experimentally the non-Markovian decoherence process.

## Methods

As one can see, the time evolution of the quantum state described by the exact master equation is fully determined by the nonequilibrium Green functions *u*(*t, t*_0_) and *v*(*t′, t*). The physical meaning of these two nonequilibrium Green functions can be seen from the following relations^34^,^35^:
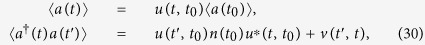
where *a* and *a*^†^ are particle annihilation and creation operators, and 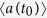 and *n*(*t*_0_) are the initial particle field and initial particle number in the system. As it has been proved in Ref. 20, the general solution of the particle propagating Green function *u*(*t, t*_0_), solved directly from Eq. (3), is given by
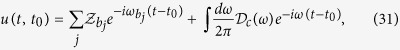
where 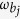 is the localized-mode frequency which is determined by 
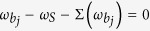
, with the corresponding amplitude 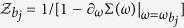, and
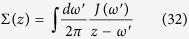


is the environment-induced self-energy correction. The dissipation-induced broadened spectrum of the system is given by Eq. (13). The solution (31) shows that the dynamics of the system contains two parts: one for the localized modes (the first term) induced by the band gaps of the spectral density, and the other one is the non-exponential damping (the second term) characterized by the dissipation spectrum 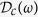, i.e. Eq (13) which is determined by the spectral density profile. The localized modes are dissipationless, a long-lived non-Markovain memory effect which can partially maintain the initial quantum coherence of the system so that the system will not evolve into classical steady state, as shown in the text.

On the other hand, the general solution of the fluctuation Green function *v*(*t*′, *t*) gives the non-equilibrium fluctuation-dissipation relation, also solved directly from Eq. (4)^20^,^34^,
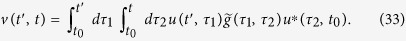
Its steady-state value is given by Eq. (16). The solution (16) gives rise to the different particle distributions for the four different steady states leading to the complexity of QCT, as shown explicitly in the text.

## Additional Information

**How to cite this article**: Xiong, H. N. *et al.* Non-Markovian Complexity in the Quantum-to-Classical Transition. *Sci. Rep.* **5**, 13353; doi: 10.1038/srep13353 (2015).

## References

[b1] Zhang W. M. & Feng D. H. Quantum non-integrability for finite systems. Phys. Rep. 252, 1 (1995).

[b2] Zurek W. H. Decoherence, einselection, and the quantum origins of the classical. Rev. Mod. Phys. 75, 715 (2003).

[b3] Deléglise S. *et al.* Reconstruction of non-classical cavity field states with snapshots of their decoherence. Nature 455, 510 (2008). 18818653 10.1038/nature07288

[b4] Myatt C. J. *et al.* Decoherence of quantum superpositions through coupling to engineered reservoirs. Nature 403, 269–273 (2000). 10659838 10.1038/35002001

[b5] Hanson R., Kouwenhoven L. P., Petta J. R., Tarucha S. & Vandersypen L. M. K. Spins in few-electron quantum dots. Rev. Mod. Phys. 79, 1217 (2007).

[b6] You J. Q. & Nori F. Atomic physics and quantum optics using superconducting circuits. Nature 474, 589 (2011). 21720362 10.1038/nature10122

[b7] Xiang Z.-L., Ashhab S., You J. Q. & Nori F. Hybrid quantum circuits: Superconducting circuits interacting with other quantum systems. Rev. Mod. Phys. 85, 623 (2013).

[b8] An J. H. & Zhang W. M. Non-Markovian entanglement dynamics of noisy continuous-variable quantum channels. Phys. Rev. A 76, 042127 (2007).

[b9] Wolf M. M., Eisert J., Cubitt T. S. & Cirac J. I. Assessing Non-Markovian Quantum Dynamics. Phys. Rev. Lett. 101, 150402 (2008) 18999575 10.1103/PhysRevLett.101.150402

[b10] Tu M. W. Y. & Zhang W. M. Non-Markovian decoherence theory for a double-dot charge qubit. Phys. Rev. B 78, 235311 (2008).

[b11] Breuer H. P., Laine E. M. & Piilo J. Measure for the Degree of Non-Markovian behavior of quantum processes in open systems. Phys. Rev. Lett. 103, 210401 (2009). 20366019 10.1103/PhysRevLett.103.210401

[b12] Chrus'cin'ski D. & Kossakowski A. Non-Markovian quantum dynamics: local versus nonlocal. Phys. Rev. Lett. 104, 070406 (2010). 20366866 10.1103/PhysRevLett.104.070406

[b13] Rivas A., Huelga S. F. & Plenio M. B. Entanglement and Non-Markovianity of quantum evolutions. Phys. Rev. Lett. 105, 050403 (2010). 20867898 10.1103/PhysRevLett.105.050403

[b14] Galve F., Pachón L. A. & Zueco D. Bringing entanglement to the high temperature limit, Phys. Rev. Lett. 105, 180501 (2010). 21231092 10.1103/PhysRevLett.105.180501

[b15] Xiong H. N., Zhang W. M., Wang X. & Wu M. H. Exact non-Markovian cavity dynamics strongly coupled to a reservoir. Phys. Rev. A 82, 012105 (2010).

[b16] Znidaric M., Pineda C. & Garcia-Mata I. Non-Markovian behavior of small and large complex quantum systems. Phys. Rev. Lett. 107, 080404 (2011). 21929150 10.1103/PhysRevLett.107.080404

[b17] Lei C. U. & Zhang W. M. Decoherence suppression of open quantum systems through a strong coupling to non-Markovian reservoirs. Phys. Rev. A 84, 052116 (2011).

[b18] Liu B. H. *et al.* Experimental control of the transition from Markovian to non-Markovian dynamics of open quantum systems. Nat. Phys. 7, 931 (2011).

[b19] Madsen K. H. *et al.* Observation of non-Markovian dynamics of a single quantum dot in a micropillar cavity. Phys. Rev. Lett. 106, 233601 (2011). 21770504 10.1103/PhysRevLett.106.233601

[b20] Zhang W. M., Lo P. Y., Xiong H. N., Tu M. W. Y. & Nori F. General Non-Markovian dynamics of open quantum systems. Phys. Rev. Lett. 109, 170402 (2012). 23215166 10.1103/PhysRevLett.109.170402

[b21] Ma T., Chen Y., Chen T., Hedemann S. R. & Yu T. Crossover between non-Markovain and Markovian dynamics induced by a hieraechical environment. Phys. Rev. A 90, 042108 (2014).

[b22] Chruściński D. & Maniscalco S. Degree of Non-Markovianity of quantum evolution. Phys. Rev. Lett. 112, 120404 (2014). 24724632 10.1103/PhysRevLett.112.120404

[b23] Cai C. Y., Yang L. P. & Sun C. P. Threshold for nonthermal stabilization of open quantum systems. Phys. Rev. A 89, 012128 (2014).

[b24] Rivas A., Huelga S. F. & Plenio M. B. Quantum non-Markovianity: characterization, quantification and detection. Rep. Prog. Phys. 77, 094001 (2014). 25147025 10.1088/0034-4885/77/9/094001

[b25] Lo P. Y., Xiong H. N. & Zhang W. M. Breakdown of Bose-Einstein distribution in photonic crystals. Sci. Rep. 5, 9423 (2015) 25822135 10.1038/srep09423 PMC4378511

[b26] Estrada A. F. & Pachón L. A. Quantum limit for driven linear non-Markovian open-quantum-systems. New J. Phys. 17, 033038 (2015).

[b27] Chen H. B., Lambert N., Cheng Y. C., Chen Y. N. & Nori F. Using non-Markovian measures to evaluate quantum master equations for photosynthesis. arXiv: 1503.02412 (2015). 10.1038/srep12753 PMC4523852 26238479

[b28] Ali M. M., Lo P. Y., Tu M. W. Y. & Zhang W. M. The short-time and long-time behaviors of Non-Markovianity measure through the two-time correlations in open quantum systems. arXiv: 1505.05748 (2015).

[b29] Feynman R. P. & Vernon F. L. The theory of a general quantum system interacting with a linear dissipative system. Ann. Phys. 24, 118 (1963).

[b30] Caldeira A. O. & Leggett A. J. Quantum tunnelling in a dissipative system. Ann. Phys. 149 374 (1983).

[b31] Haake F. & Reibold R. Strong damping and low-temperature anomalies for the harmonic oscillator. Phys. Rev. A 32, 2462 (1985). 9896361 10.1103/physreva.32.2462

[b32] Karrlein R. & Grabert H. Exact time evolution and master equations for the damped harmonic oscillator. Phys. Rev. E 55, 153 (1997).

[b33] Zhang W. M., Feng D. H. & Gilmore R. Coherent states: theory and some applications. Rev. Mod. Phys. 62, 867 (1990).

[b34] Jin J. S., Tu M. W. Y., Zhang W. M. & Yan Y. J. Non-equilibrium quantum theory for nanodevices based on the Feynman-Vernon influence functional. New J. Phys. 12, 083013 (2010).

[b35] Lei C. U. & Zhang W. M. A quantum photonic dissipative transport theory. Ann. Phys. 327, 1408 (2012).

[b36] Haug H. & Jauho A.-P. Quantum Kinetics in Transport and Optics of Semiconductors, 2nd Ed. (Springer Series in Solid-State Sciences 123, Berlin, 2007).

[b37] Lambropoulos P., Nikolopoulos G. M., Nielsen T. R. & Bay S. Fundamental quantum optics in structured reservoirs. Rep. Prog. Phys. 63, 455 (2000).

[b38] Schwinger J. Brownian Motion of a Quantum Oscillator. J. Math. Phys. 2, 407 (1961).

[b39] Keldysh L. V. Diagram Technique for Nonequilibrium Processes. Sov. Phys. JETP 20, 1018 (1965).

[b40] Kadanoff L. P. & Baym G. Quantum Statistical Mechanics (Benjamin, New York, 1962).

[b41] Anderson P. W. Absence of Diffusion in Certain Random Lattices. Phys. Rev. 109, 1492 (1958).

[b42] Anderson P. W. Localized magnetic states in metals. Phys. Rev. 124, 41 (1961).

[b43] Fano U. Effects of configuration interaction on intensities and phase shift. *Phys*. Rev. 124, 1866 (1961).

[b44] Mahan G. D. Many-Body Physics, 3rd Ed. (Kluwer Academic/Plenum Publishers, New Yoek, 2000), p.207-208

[b45] Friedrichs K. O. On the perturbation of continuous spectra. Commun. Pure Appl. Math. 1, 361 (1948).

[b46] Lee T. D. Some special examples in renormalizable field theory. Phys. Rev. 95, 1329 (1954).

[b47] Prigogine I. Dissipative processes in quantum theory. Phys. Rep. 219, 93 (1992).

[b48] Leggett A. J. *et al.* Dynamics of the dissipative two-state system. Rev. Mod. Phys. 59, 1 (1987).

[b49] Kubo R. The fluctuation-dissipation theorem. Rep. Prog. Phys. 29, 255 (1966).

[b50] Langevin P. On the Theory of Brownian Motion. C. R. Acad. Sci. (Paris) 146, 530 (1908).

[b51] Pachón L. A. & Brumer P. Direct experimental determination of spectral densities of molecular complexes. J. Chem. Phys. 141, 174102 (2014) and references therein. 25381497 10.1063/1.4900512

[b52] Ali M. M., Lo P. Y. & Zhang W. M. Exact decoherence dynamics of 1/f noise. New. J. Phys. 16, 103010 (2014) and references therein.

[b53] Paavola J., Piilo J., Suominen K.-A. & Maniscalco S. Environment-dependent dissipation in quantum Brownian motion. Phys. Rev. A 79, 052120 (2009) and references theein.

[b54] Wu M. H., Lei C. U., Zhang W. M. & Xiong H. N. Non-Markovian dynamics of a microcavity coupled to a waveguide in photonic crystals. Opt. Exp. 18, 18407 (2010). 10.1364/OE.18.018407 20721235

[b55] Pachón L. A. Triana J. F., Zueco D. & Brumer P. Uncertainty principle consequences at thermal equilibrium, arXiv:1401.1418.

[b56] Grabert H., Schramm P. & Ingold G.-L. Quantum Brownian motion: the functional integral approach. Phys. Rep. 168, 115 (1988).

[b57] Bachor H.-A. & Ralph T. C. A Guide to Experiments in Quantum Optics (Wiley-VCH), 2nd ed, (2004).

[b58] Yang P. Y., Lin C. Y. & Zhang W. M. Transient current-current correlations and noise spectra. Phys. Rev. B 89, 115411 (2014).

